# Current status and influencing factors of nurses’ work engagement in Chinese tertiary hospitals: A latent profile analysis

**DOI:** 10.1371/journal.pone.0321398

**Published:** 2025-04-03

**Authors:** Xiaoli Zhong, Xian Rong, Yuxin Li, Wei Qing, Guiqiong Xie, Ping Dai, Jijun Wu, Lin He

**Affiliations:** 1 Department of Nursing, Deyang People’s Hospital, Sichuan, China; 2 Sichuan Nursing Vocational College, Sichuan, China; 3 School of Nursing, North Sichuan Medical College, Sichuan, China; 4 Department of Cardiology, Deyang People’s Hospital, Sichuan, China; University of Luzon, PHILIPPINES

## Abstract

**Background:**

Work engagement is a work-related state of mind full of positivity and vigor. Understanding the current status and influencing factors of nurses’ work engagement is essential for improving the quality of nursing services and stabilizing the nursing workforce building. This study aimed to explore the potential categories of nurses’ work engagement and their influencing factors and provide a reference basis for developing targeted interventions to improve their work engagement.

**Methods:**

From March to April 2024, 1,919 nurses from 12 tertiary hospitals in Sichuan Province, China, were enrolled in the study using convenience sampling. A demographic profile questionnaire, work engagement scale, and professional mission scale were used to investigate them. Latent profile analysis was used to explore the categories of nurses’ work engagement, and unordered multicategorical logistic regression was used to analyze the influencing factors of each category.

**Results:**

Nurses’ work engagement could be categorized into three potential profiles: low work engagement group (n = 659, 34.3%), medium work engagement group (n = 763, 39.8%), and high work engagement group (n = 497, 25.9%). The unordered multi-categorical logistic regression results showed that marital status, reasons for choosing a nursing specialty, self-rated sleep quality, current work intensity, and sense of professional mission were influential factors affecting the potential profile of work engagement among nurses in tertiary care hospitals. Among them, unmarried nurses were more likely to belong to the low work engagement group; those who chose nursing specialties based on personal interest were more likely to belong to the medium work engagement group; those with medium work intensity were more likely to belong to the high work engagement group; and those with good self-assessed sleep quality and higher scores of sense of professional mission were more likely to belong to the medium and high work engagement groups.

**Conclusion:**

The potential profiles of nurses’ work engagement in Chinese tertiary hospitals were dominated by the medium and low work engagement groups, with significant heterogeneity. Nursing managers should tailor interventions to enhance nurses’ sense of professional mission according to the characteristics of each type of nurse work engagement, thereby improving work engagement and nursing service quality.

## Introduction

With the rapid development of health care and the aging of the population, the demand for quality nursing services is increasing. Nurses are critical in providing healthcare services and essential to healthcare organizations. However, the shortage of nursing human resources and high turnover have become important public health issues globally [1]. According to WHO data, it is expected that by 2030, the global shortage of nurses will be as high as 5.7 million [2]. Due to the unique nature of nursing work, nurses face high-intensity, high-pressure, high-load work environments for a long time. They are prone to burnout and willingness to leave, resulting in leaving behavior and a more significant shortage of nursing human resources. The shortage of nursing human resources and the high turnover rate will increase the work pressure of in-service nursing staff, leading to burnout, weakening nurses’ motivation, reducing nurses’ commitment to their work, and affecting the quality of nursing services [3].

Work engagement was proposed by Kahn in 1990 and is currently a research hotspot in positive psychology. Foreign scholars Schaufeli defined it as a lasting, fulfilling, and positive emotional and cognitive state, reflecting the degree of vitality, dedication, and concentration shown by individuals at work, which most scholars at home and abroad now recognize [4]. Among them, vitality indicates that individuals have abundant energy, high enthusiasm, and a positive mindset at work; dedication indicates their commitment, loyalty, and willingness to pay for their work; and focus indicates their devotion and concentration to their work. On the one hand, from the organizational level, a high level of work dedication can effectively enhance employees’ work efficiency, promote organizational commitment, and improve work performance. On the other hand, from the individual level, a high level of work engagement is conducive to enhancing employees’ job satisfaction and reducing employees’ burnout and turnover behavior [5]. Previous studies have shown that foreign nurses’ work commitment is at a moderate level due to a variety of factors, and about 15% of nurses have low work commitment [6]. In addition, studies have also shown that Chinese nurses’ work engagement is at a medium or medium-low level, which is lower than that of foreign nurses [7,8]. Nurses’ work engagement is related to various influencing factors, such as individual, organizational, and social environment factors [9–11]. In addition, several studies have confirmed that nurses’ work engagement positively impacts patient satisfaction, organizational commitment, and job performance [12,13]. Therefore, exploring the current situation and influencing factors of nurses’ work engagement is of great significance for improving the quality of nursing services and stabilizing the construction of the nursing team.

The sense of professional mission refers to an individual’s deep sense of responsibility and value for the work or profession they are engaged in, as well as identification with and commitment to the goals and meaning of the work they are involved in, which is mainly manifested in altruistic behaviors, orienting power, and proactive and aggressive. The Job Demand-Resource Model states that when an employee has a higher level of personal resources, it positively affects the individual’s career development [14]. Research also shows that a sense of professional mission is a vital personal resource; nurses with a high sense of professional mission can find the meaning and value of their work amid complicated work, experience the sense of fulfillment and happiness brought by their profession, and are more willing to give their time and energy to devote to their work [15]. Research shows that nurses’ sense of professional mission can positively predict their level of work engagement, i.e., the higher the nurses’ sense of professional mission, the higher the level of work engagement. According to the theory of career construction, which mainly emphasizes the individual’s self-realization and value identity at work, it is believed that work must be compatible with the individual’s core values and mission to obtain the most excellent satisfaction and sense of achievement [16]. Nurses with a strong sense of mission are more likely to be driven by intrinsic motivation, able to focus more on their work, and willing to invest more time and energy in their work, thus receiving positive feedback at work and forming a virtuous cycle.

Current studies on professional mission and work engagement have used a variable-centered approach to explore the influencing factors through unifactorial, multifactorial, and correlation analyses. When considering the heterogeneity of work engagement, it is unknown whether the factors affect work engagement in different categories of nurses. Latent profile analysis is a research methodology centered around individuals that identify subgroups of variables with similar relationships or levels of similarity, and its classification accuracy is significantly better than traditional classifications, helping to visualize the proportions of different categories and population characteristics [17]. As an important part of healthcare organizations, the level of work engagement of nurses in tertiary hospitals plays an important role in deepening quality nursing services and promoting high-quality nursing development. Given that there are fewer studies on the relationship between work engagement and a sense of professional mission among nurses in tertiary hospitals in China, the present study intends to explore the potential categories of work engagement among nurses in tertiary hospitals based on potential profiling and explore the group differences and influencing factors of different categories, to provide a basis for the development of targeted interventions for nurses in different work engagement categories.

## Method

### Study design

This study is a cross-sectional study. The design of this study followed the guidelines for reporting observational studies (STROBE).

### Participants

An anonymous online questionnaire was administered to nurses in a total of 12 tertiary hospitals in Sichuan Province from March to April 2024.Convenience sampling was used to select two tertiary hospitals from each of the six regions of Sichuan Province (East Sichuan, West Sichuan, South Sichuan, North Sichuan, Central Sichuan, and Chengdu). All tertiary hospitals selected for this study had more than 1,000 beds and more than 800 clinical nurses. Inclusion criteria: (1) secondary school education or above, clinical internship of 8 months or above, and passing the nursing qualification examination and obtaining the nursing qualification certificate; (2) working in clinical nursing for more than 2 years; (3) informed consent and voluntary participation in this study. Exclusion criteria: (1) internship, regulation training, and continuing education nurses; (2) absent nurses who were on maternity leave, personal leave, sick leave, and so on. A total of 2032 questionnaires were recovered in this study, and 113 invalid questionnaires with logical errors and regular responses were excluded, resulting in a total of 1919 valid questionnaires, with a valid recovery rate of 94.4%.

### Sample size

According to Kendall’s sample size estimation method [18], the sample size is generally 5–10 times the number of study variables. This study included demographic variables (16), work engagement (3 dimensions), and sense of professional mission (3 dimensions) of the study subjects, totaling 22 variables, and considering 20% of invalid questionnaires, the sample size should be calculated as 132–264. In addition, the potential profiling analysis requires the sample size to be greater than 500, and 1,919 research subjects were included in this study, which meets the above requirements.

### Data collection

The questionnaires were created and distributed using Questionstar software. After obtaining the consent of the director of the nursing department of all the surveyed hospitals, a person in charge of the survey study was selected in each hospital, and all the persons in charge were given unified training to inform them of the purpose, significance, and precautions of this survey study. The person in charge sent the link to the questionnaire to all the nurses’ groups in the hospitals, and the participants completed it on their own and submitted it online. To ensure the completeness of the questionnaire, all options were set as mandatory questions. To avoid duplication, each IP address could only be entered once. At the end of the survey, the collected questionnaires were evaluated, and questionnaires with apparent regularity of completion and logical errors were excluded.

### Measures

#### Sociodemographic characteristics.

The researcher designed the general demographic information for this study after reviewing the literature, including gender, age, marital status, professional title, position, self-assessed sleep quality, and current work intensity, etc.

Among them, the title refers to a kind of identification and evaluation of the professional level and ability of nursing personnel in the field of nursing specialization, mainly including Nurse(the initial level of nursing title, usually awarded to the personnel who have just completed secondary professional education in nursing and have a good command of the knowledge and skills of basic nursing care and general specialized nursing care), Nurse Practitioner (advanced title in the title system of nurses, usually refers to the nurses with college or university education who have been working for three to five years, or who have obtained the qualification of nurse practitioner by examination). or those who have obtained the qualification of nurse practitioner by examination. In addition to basic nursing work, they are also required to have certain teaching and scientific research abilities), Nurse Supervisor (intermediate professional title of nursing personnel, requiring a wealth of professional knowledge and clinical experience in nursing, and proficiency in common first aid and nursing technical operations), Deputy chief nurse and above (deputy senior professional title of nursing personnel, requiring a high level of professional competence and rich clinical experience).

Positions refer to the specific positions and job responsibilities held by nurses in a healthcare organization and include Clinical Nurse (those responsible for direct patient care), Nursing team leader (those responsible for nursing leadership of a unit or department), and Head Nurse (those responsible for nursing leadership of a particular district within a hospital).

Self-assessment of sleep quality: Good (Adequate sleep, usually between 7 and 9 hours, full of energy and concentration during the day, high work efficiency); General (Sleep between 6 and 7 hours, although slightly insufficient, daytime energy is still good, but there may be a brief period of drowsiness); Worse (Sleeping less than 6 hours, feeling sleepy and tired during the day, lack of concentration).

Current work intensity: Low (Average daily working hours ≤ 8 hours and night shift frequency < 3 times/month); Middle (Average daily working hours > 8 hours or night shift frequency ≥ 3 times/month); High (Average daily working hours > 8 hours and night shift frequency ≥ 3 times/month).

#### Work engagement scale.

This scale was developed by Schaufeli et al [19]. in 2006 and Chineseized by Li et al [20]. in 2013. The scale contains Concentration (3 entries), Devotion (3 entries), and Vigor (3 entries). A 7-point Likert scale was used, with a total score of 0–54, and the higher the score, the higher the level of commitment to their work. The scale has good reliability and validity with a Cronbach alpha coefficient of 0.933. In the present study, the Cronbach alpha coefficient for the scale was 0.945.

#### Professional mission scale.

The scale was compiled by Dobrow et al [21]. in 2011 and Chineseized by Zhang et al [22]. in 2015. The scale consists of 3 dimensions, namely orientation, charitable contribution, meaning, and value, with ten entries. A 5-point Likert scale was used, with a total score of 11–55, with higher scores indicating a higher sense of professional mission. The scale has good reliability and validity with a Cronbach alpha coefficient of 0.909. The Cronbach alpha coefficient for the scale in this study was 0.969.

### Ethical consideration

This study followed the Declaration of Helsinki and was approved by the Ethics Committee of Deyang People’s Hospital (2024-04-016-K01). At the beginning of the anonymous survey, an informed consent form was included on the cover of the online questionnaire, and completion and submission of the questionnaire was considered as informed consent and voluntary participation in this survey. All participants consciously and voluntarily agreed to participate in this survey.

### Statistical analysis

Mplus 8.3 was used for potential profile analysis. The nine entries of the work engagement scale were used as exogenous variables. The number of possible profiles was increased sequentially to determine the optimal model and name its classification. The model fit evaluation indexes were divided into three categories: ① Information indexes were Aicheck Information Criterion (AIC), Bayesian Information Criterion (BIC), and Adjusted Bayesian Information Criterion (aBIC), with smaller values indicating better fit; ② Likelihood Ratio Test indexes were Corrected Likelihood Ratio Test (LMR) and Bootstrap-based Likelihood Ratio Test (BLRT), and when *P* <  0.05 indicated that the When *P* <  0.05, it suggests that the k-category model is better than the k-1 category model; ③ The classification index is entropy, whose value ranges from 0 to 1, and the closer it is to 1, the more accurate the classification is. Data were analyzed using SPSS 26.0 software, and comparisons between groups were made using the χ2 test and ANOVA. Logistic regression was used to analyze the factors influencing the potential profile of nurses’ work engagement.

## Results

### Potential profile analysis of nurses’ work engagement

Using the nine entries of the work engagement scale as exogenous variables, one to five potential profile models were fitted sequentially, and the model fitting information is shown in Table 1. The results show that as the number of models increases, the values of AIC, BIC, and aBIC decrease, but the LMR of model 4 does not reach a significant level. Values did not reach the significant level; the fitting results of model 5 were qualified, but the classification needed to be simplified and lacked practical clinical significance; model 3 was satisfactory in all indicators and lacked clinical importance. Model 3 was ideal in all indicators, with an entropy value of 0.942, and the average probability of belonging to the three potential categories was 96.8%-98.5%, indicating that the discriminative power of the model was more accurate, see Table 2. Considering the fit’s evaluation indicators and the categorization’s practical significance, model 3 was selected as the best-fitting model.

**Table 1:** Indicators for fitting the potential profile model of nurses’ work engagement.

Model	AIC	BIC	aBIC	Entropy	*P*-value		Category probability(%)
					LMR	BLRT	
1	46920.425	47020.497	46963.311	–	–	–	–
2	37907.202	38062.870	37973.913	0.934	<0.001	<0.001	50.4/49.6
3	34156.232	34367.495	34246.768	0.942	<0.001	<0.001	34.3/39.8/25.9
4	31003.942	31270.801	31118.305	1	0.143	<0.001	5.7/33.4/32.3/28.6
5	29944.401	30266.856	30082.589	0.966	<0.001	<0.001	7.8/25.1/29.9/23.3/13.9

**Table 2:** Probability matrix for attribution of 3 potential profile types for nurses’ work engagement.

Potential profile type	Probability of attribution to potential category (%)		
	C1	C2	C3
1	0.973	0.027	0.000
2	0.022	0.968	0.009
3	0.000	0.015	0.985

**Fig 1:**
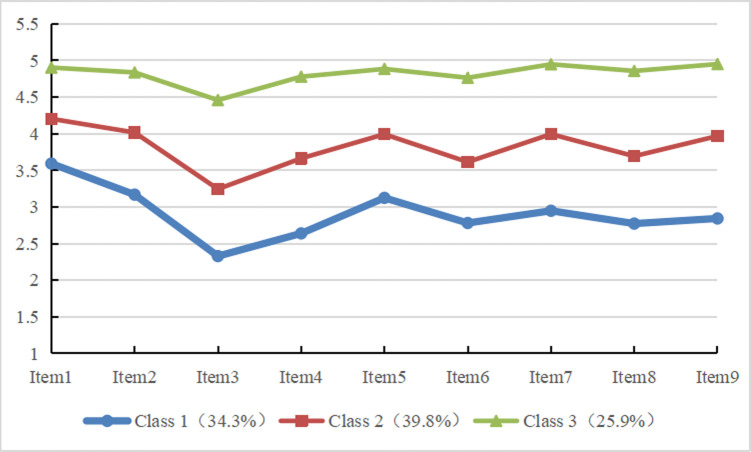
Overview of potential profiles of nurses’ work engagement.

The chart is drawn according to the scores of 9 entries in 3 categories of nurses’ work engagement. See Fig 1. According to the scoring characteristics of each category, category 1 nurses scored significantly lower than other categories, so it was named “low work engagement group”, with a total of 659 nurses (34.3%). Category 2 nurses scored between C1 and C3 in each item, and the overall score was in the medium level, so it was named “medium work engagement group”, with a total of 763 nurses (39.8%). Category 3 nurses scored significantly higher in all items than other categories, so it was named “high work engagement group”, with a total of 497 nurses (25.9%).

### Univariate analysis of potential categories of nurses’ work engagement

The results of the univariate analysis showed that the three groups of nurses were statistically significant (*P* < 0.05) in terms of marital status, highest education, title, position, years of work experience, average monthly personal income, whether they worked night shifts or not, reasons for choosing the nursing specialty, self-assessed health status, self-assessed quality of sleep, current work intensity, and scores of sense of professional mission, as shown in Table 3.

**Table 3:** Univariate analysis of potential categories of nurses’ work engagement.

Variables		C1 (n = 659)	C2 (n = 763)	C3 (n = 497)	χ^2^/*F*	*P*
Age	<30	390 (59.2%)	333 (43.6%)	193 (38.8%)	68.165	<0.001
	30~<40	226 (34.3%)	323 (42.3%)	216 (43.5%)		
	≥40	43 (6.5%)	107 (14.0%)	88 (17.7%)		
Gender	Male	44 (6.7%)	31 (4.1%)	29 (5.8%)	4.939	0.085
	Female	615 (93.3%)	732 (95.9%)	468 (94.2%)		
Marital or childbearing status	Unmarried	224 (34.0%)	180 (23.6%)	90 (18.1)	48.827	<0.001
	Married with no children	81 (12.3%)	77 (10.1%)	51 (10.3%)		
	Married with children	338 (51.3%)	484 (63.4%)	336 (67.6%)		
	Divorced/widowed	16 (2.4%)	22 (2.9%)	20 (4.0%)		
Highest education	Junior college and below	182 (27.6%)	146 (19.1%)	123 (24.7%)	17.865	0.001
	Undergraduate	468 (71.0%)	606 (79.4%)	372 (74.8%)		
	Master degree or above	9 (1.4%)	11 (1.4%)	2 (0.4%)		
Department	Internal medicine system	192 (29.1%)	208 (27.3%)	138 (27.8%)	16.086	0.187
	Surgical system	165 (25.0%)	177 (23.2%)	127 (25.6%)		
	Maternal and child system	91 (13.8%)	116 (15.2%)	70 (14.1%)		
	Critical care system	52 (7.9%)	56 (7.3%)	18 (3.6%)		
	Operating room systems	29 (4.4%)	46 (6.0%)	31 (6.2%)		
	Outpatient and emergency system	56 (8.5%)	77 (10.1%)	58 (11.7%)		
	Otherwise	74 (11.2%)	83 (10.9%)	55 (11.1%)		
Professional title	Nurse	142 (21.5%)	117 (15.3%)	77 (15.5%)	48.687	<0.001
	Nurse Practitioner	316 (48.0%)	300 (39.3%)	195 (39.2%)		
	Nurse Supervisor	187 (28.4%)	291 (38.1%)	195 (39.2%)		
	Deputy chief nurse and above	14 (2.1%)	55 (7.2%)	30 (6.0%)		
Position	Clinical Nurse	632 (95.9%)	696 (91.2%)	451 (90.7%)	18.725	0.001
	Nursing team leade	25 (3.8%)	64 (8.4%)	40 (8.0%)		
	Head Nurse	2 (0.3%)	3 (0.4%)	6 (1.2%)		
Type of contract	Labor contract	585 (88.8%)	649 (85.1%)	419 (84.3%)	5.967	0.051
	Professional preparation	74 (11.2%)	114 (14.9%)	78 (15.7%)		
Years of experience	≤3	133 (20.2%)	107 (14.0%)	63 (12.7%)	60.821	<0.001
	4~<10	279 (42.3%)	257 (33.7%)	158 (31.8%)		
	10~<15	173 (26.3%)	242 (31.7%)	149 (30.0%)		
	≥15	74 (11.2%)	157 (20.6%)	127 (25.6%)		
Average monthly personal income	<5000	250 (37.9%)	197 (25.8%)	140 (28.2%)	44.956	<0.001
	5000~<8000	323 (49.0%)	385 (50.5%)	237 (47.7%)		
	8000~<11000	70 (10.6%)	139 (18.2%)	90 (18.1%)		
	≥11000	16 (2.4%)	42 (5.5%)	30 (6.0%)		
Whether on night shift	Yes	499 (75.7%)	484 (63.4%)	286 (57.5%)	45.891	<0.001
	No	160 (24.3%)	279 (36.6%)	211 (42.5%)		
Reasons for choosing the nursing profession	Personal interest	87 (13.2%)	200 (26.2%)	187 (37.6%)	98.594	<0.001
	Teacher/Family Recommendations	465 (70.6%)	483 (63.3%)	268 (53.9%)		
	Specialized transfers	107 (16.2%)	80 (10.5%)	42 (8.5%)		
Specialty Nurse Certification	No	541 (82.1%)	589 (77.2%)	385 (77.5%)	6.398	0.171
	Provincial Nurse Specialist	104 (15.8%)	157 (20.6%)	99 (19.9%)		
	National Specialized Nurses	14 (2.1%)	17 (2.2%)	13 (2.6%)		
Self-assessed health status	Good	293 (44.5%)	509 (66.7%)	385 (77.5%)	158.269	<0.001
	General	315 (47.8%))	240 (31.5%)	104 (20.9%)		
	Worse	51 (7.7%)	14 (1.8%)	8 (1.6%)		
Self-assessment of sleep quality	Good	134 (20.3%)	282 (37.0%)	264 (53.1%)	168.445	<0.001
	General	314 (47.6%)	356 (46.7%)	178 (35.8%)		
	Worse	211 (32.0%)	125 (16.4%)	55 (11.1%)		
Current work intensity	Low	2 (0.3%)	8 (1.0%)	12 (2.4%)	73.305	<0.001
	Middle	314 (47.6%)	478 (62.6%)	337 (67.8%)		
	High	343 (52.0%)	277 (36.3%)	148 (29.8%)		
Total score of professional mission		32.00 ± 4.88	40.84 ± 4.16	48.69 ± 2.63	165.088	<0.001

### Multifactorial analysis of potential categories of nurses’ work engagement

Taking the potential category of nurses’ work engagement as the dependent variable (“low work engagement group” as the reference) and the statistically significant factors in the univariate analysis as the independent variables, the parallel line test showed that *P <* 0.001, which did not meet the conditions of ordered regression analysis, so we constructed an unordered multi categorical logistic regression model to conduct multifactorial analysis. The results of the study showed that marital status, reasons for choosing the nursing profession, self-rated sleep quality, current work intensity, and sense of professional mission were influential factors affecting the potential profile of nurses’ work engagement (all *P* < 0.05). The results of the unordered multi-categorical logistic regression are shown in Table 4. unmarried were more likely to belong to the low work engagement group (OR = 0.115, *P* = 0.006); choosing a nursing specialty based on personal interest was more likely to belong to the medium work engagement group (OR = 1.835, *P* = 0.047); and good self-rated sleep quality was more likely to belong to the medium and high work engagement groups (OR = 1.768, *P* = 0.030; OR = 2.547, *P* = 0.020); moderate work intensity was more likely to belong to the high work engagement group (OR = 1.993, *P* = 0.008); and higher scores of the professional mission were more likely to belong to the moderate and high work engagement group (OR = 1.534, *P* < 0.001; OR = 2.591, *P* < 0.001).

**Table 4:** Multifactorial analysis of potential categories of nurses’ work engagement.

Variables		C2 VS. C1				C3 VS. C1			
		*β*	OR	95%CI	P	*β*	OR	95%CI	P
Marital or childbearing status	Unmarried	–0.588	0.556	(0.190, 1.627)	0.284	–2.163	0.115	(0.024, 0.544)	0.006
	Married with no children	–0.573	0.564	(0.183, 1.740)	0.319	–1.401	0.246	(0.050, 1.224)	0.087
	Married with children	–0.337	0.714	(0.263, 1.939)	0.509	–1.167	0.311	(0.076, 1.268)	0.103
Reasons for choosing the nursing profession	Personal interest	0.607	1.835	(1.008, 3.339)	0.047	0.343	1.409	(0.108, 3.886)	0.451
	Teacher/Family Recommendations	0.375	1.454	(0.888, 2.382)	0.137	0.176	1.193	(0.089, 3.182)	0.662
Self-assessment of sleep quality	Good	0.570	1.768	(1.057, 2.960)	0.030	0.935	2.547	(1.161, 5.589)	0.020
	General	0.324	1.382	(0.900, 2.124)	0.140	0.389	1.476	(0.729, 2.990)	0.280
Current work intensity	Low	1.426	4.161	(0.429, 40.361)	0.219	1.890	6.621	(0.480, 91.377)	0.158
	Middle	0.335	1.398	(0.996, 1.963)	0.053	0.689	1.993	(1.193, 3.327)	0.008
Total score of professional mission		0.428	1.534	(1.467, 1.603)	<0.001	0.952	2.591	(2.413, 2.782)	<0.001

## Discussion

This study found that the work engagement of nurses in Chinese tertiary hospitals could be categorized into three potential profiles through potential profile analysis: low work engagement group (34.3%), medium work engagement group (39.8%), and high work engagement group (25.9%), which indicated that there was group heterogeneity in the work engagement of nurses in tertiary hospitals. Among them, nurses in the low work engagement group were mostly < 30 years old, with average self-assessed health, average self-assessed sleep quality, and high work intensity; nurses in the medium work engagement group were mainly with average self-assessed sleep quality and medium work intensity; and nurses in the high work engagement group were mainly with age 30 to < 40 years old, good self-assessed health, and good self-assessed sleep quality. The nurses in the medium-low work engagement group accounted for 74.1%, indicating that the work engagement of tertiary nurses was at a medium-low level, which was lower than the findings of relevant studies by Spanish scholars [23,24]. Analyzing the reasons, on the one hand, the shortage of nursing human resources is a common problem in the medical industry, which is especially prominent in tertiary hospitals. The respondents in this study were nurses in tertiary hospitals. Nurses in tertiary hospitals often face high-intensity and high-load workloads. The working environment and interpersonal relationships are more complex. The prolonged work pressure will lead to physical and mental fatigue and burnout, which will decrease nurses’ work engagement. In addition, as some tertiary hospitals may have inadequate compensation and promotion mechanisms, the lack of sufficient incentives can lead to a lack of motivation, thus affecting the nurses’ work engagement [25]. Therefore, nursing managers should optimize the allocation of human resources to reduce nurses’ work pressure and improve the incentive mechanism to create a supportive work environment for nurses to stimulate nurses’ work enthusiasm and work engagement.

This study found that unmarried nurses were more likely to belong to the low work engagement group, similar to the results of previous study by Turkish scholar [26]. Analyzing the reasons, on the one hand, unmarried nurses mostly have lower working years, relatively lack of professional ability and working experience, and young nurses may not yet have a clear direction for their career development, and their knowledge of the nursing profession is not yet clear enough, which affects their level of work engagement [27]; on the other hand, unmarried nurses may face more personal pressures and uncertainties, such as career planning, family expectations, and marital problems, that can distract them from their work. However, some related studies have also found that married nurses have a lower level of work engagement, which may be related to the fact that married nurses will devote more time and energy to their families, thus affecting their work engagement [9]. Therefore, it is suggested that nursing managers should pay attention to the marital status of nurses, focus on the individualized situation of nurses, pay attention to the personal development needs of nurses, and help nurses to balance their work and personal life to enhance their overall job satisfaction and engagement level.

This study found that choosing a nursing program based on personal interest was more likely to fall into the moderate work engagement group. Self-determination theory emphasizes that individuals feel more satisfied and happy when engaging in activities consistent with their interests and values [28]. On the one hand, nurses who chose nursing specialties based on personal interests were more motivated by their inner love and interest than by external rewards or pressures. This inner drive can make nurses more resilient and motivated when facing work challenges [29]. On the other hand, when nurses choose a nursing career due to their interests, they are often willing to invest time and energy to learn new knowledge and skills to improve their professional abilities continuously. This continuous learning and growth enhances one’s professional competitiveness, increases the sense of accomplishment at work, and further enhances work engagement. Therefore, it is suggested that nursing managers should focus on cultivating nurses’ love for the nursing profession, emphasizing the vital contribution of nursing to patients’ health and social well-being, enhancing nurses’ sense of professional honor, and stimulating nurses’ professional interests to improve work engagement.

This study found that nurses with moderate work intensity had higher levels of work engagement. In comparison, nurses with high work intensity had lower levels of work engagement, which is consistent with the results of several studies [30,31]. Due to the unique nature of nursing work, nurses often face high load and high-intensity workloads, and long-term high-intensity work will lead to physical fatigue, which not only affects the physical and mental health of nurses but may increase the risk of medical errors. Foreign studies have shown that nurses’ work intensity increases, their work engagement decreases, and the incidence of needlestick injuries increases by 35% [32]. In addition, high-stress levels often accompany high-intensity work, and prolonged exposure to high stress may lead to burnout, emotional exhaustion, depersonalization (apathetic attitudes toward patients), and a decreased sense of personal accomplishment, which affects nurses’ job satisfaction and reduces their work efficiency and work engagement [33]. Therefore, nursing managers should create a supportive work environment for nurses, reasonably control work intensity, ensure reasonable distribution of work tasks, and provide adequate support and resources to avoid overwork and burnout.

The results of this study showed that nurses with good self-assessed sleep quality were more likely to be categorized in the moderate and high work engagement group, similar to previous study by Austrian scholar [34]. According to Maslow’s hierarchy of needs theory, sleep is one of the most critical ways to satisfy basic human physiological needs. It is vital in maintaining internal balance and promoting physical and mental health. Several studies at home and abroad have pointed out that the prevalence of sleep disorders and poor sleep quality among nurses is 39.2% ~  63.5% [35,36], with the problem of sleep disorders among shift nurses being particularly prominent [37]. Long-term sleep deprivation by nurses may lead to endocrine disorders and increase the risk of obesity, hypertension, coronary heart disease, and other diseases on the one hand.On the other hand, it may also lead to individual memory loss, slow thinking, anxiety, depression, and other negative emotions, which affect the physical and mental health of nurses and their work status and then lead to presenteeism and reduced work engagement [38,39]. Resource conservation theory also points out that human beings tend to protect their resources and avoid loss of resources when coping with stress and challenges [40]. Sleep, as a vital physiological and psychological resource, directly impacts work engagement. Adequate sleep helps restore and rebuild nurses’ physical and emotional resources, facilitating nurses’ active engagement in their work and improving work efficiency and quality of care. Therefore, it is recommended that nursing managers pay attention to developing nurses’ physical and mental health, rationalizing nurses’ shifts, avoiding long shifts, and ensuring sufficient sleep to devote themselves to their work in a better state.

This study found that nurses’ sense of professional mission in tertiary hospitals is at a medium level. The higher the score of the professional mission, the more likely it is to belong to the group of medium and high work engagement. Previous studies have also confirmed that the sense of professional mission is essential to nurses’ work engagement [41]. As a form of internalization of professional values, forming a professional mission is a long-term dynamic process. Nurses’ sense of professional mission is the degree of nurses’ recognition of and concern for their work tasks and responsibilities, including concern for the health and well-being of patients, love for nursing work, and recognition of the nursing profession, which drives individuals to devote themselves to their work. Self-determination theory points out that the individual’s behavioral motivation results from the interaction between intrinsic needs and extrinsic environmental factors and emphasizes the influence of autonomy, competence, and motivation on the individual’s behavioral motivation [42]. The sense of professional mission stems precisely from the inner love of something and is an internal motivation with a high degree of automation and self-determination. When nurses feel that their work is meaningful and valuable and can contribute to society and others, their needs for autonomy and competence are satisfied, and this satisfaction will stimulate their intrinsic motivation, making them love their work more and be willing to put more time and energy into it, so the level of work engagement is higher [29]. Therefore, nursing managers should pay attention to and cultivate nurses’ sense of professional mission, encourage the opening of educational courses related to the sense of professional mission, carry out in-depth publicity and education on the meaning of the profession in daily work, and clarify the vision and values of the nursing profession. In addition, nursing managers should create a supportive working environment for nurses, encourage nurses to participate in departmental management and decision-making, authorize them appropriately, and give them regular feedback and recognition for their work to increase their sense of work autonomy and promote their inner sense of mission with a sense of independence to stimulate the nurses’ work engagement.

## Limitations

This study still has some limitations. First, this study is a cross-sectional study, and the causal relationship between the independent and dependent variables cannot yet be clarified; second, this study only investigated 12 tertiary hospitals in Sichuan Province, China, and has not yet investigated other levels of hospitals, with a limited sample size and limited generalization and extrapolation of the findings; it is suggested that longitudinal, multicenter, and large-sample investigations can be carried out in the future, as well as corresponding intervention studies, in order to further validate and improve the conclusions of this study.

## Conclusion

This study analyzed the potential profiles of nurses’ work input and their influencing factors in tertiary hospitals in Sichuan Province, China, using potential profile analysis. Nurses’ work engagement in tertiary hospitals could be categorized into low work engagement group (34.3%), medium work engagement group (39.8%), and high work engagement group (25.9%). The results of unordered multi-categorical logistic regression showed that marital status, reasons for choosing a nursing specialty, self-rated sleep quality, current work intensity, and sense of professional mission were influential factors affecting the potential profile of nurses’ work engagement in tertiary hospitals. Nursing managers should pay attention to the level of nurses’ work engagement, develop targeted interventions based on the characteristics of different categories of nurses and their influencing factors, and improve the sense of professional mission of nurses in tertiary care hospitals, which in turn improves work engagement and the quality of nursing services.

## Supporting information

S1 Data Raw data. (XLSX)
